# 959. A Gap of Knowledge: Patient Perspectives and Barriers to Sunscreen Use After Burn Injury

**DOI:** 10.1093/jbcr/irag033.537

**Published:** 2026-04-06

**Authors:** Sarah Wang, Dania B Johnson, Elizabeth Flores, Karel-Bart Celie, Katrina Lee, T Justin Gillenwater, Maxwell B Johnson, Haig A Yenikomshian

**Affiliations:** Keck School of Medicine, University of Southern California, Los Angeles, California; Keck School of Medicine, University of Southern California, Los Angeles, California; Keck School of Medicine, University of Southern California, Los Angeles, California; Division of Plastic and Reconstructive Surgery, Keck School of Medicine, University of Southern California, Los Angeles, California; Keck School of Medicine, University of Southern California, Los Angeles, California; Division of Plastic and Reconstructive Surgery, Keck School of Medicine, University of Southern California, Los Angeles, California; Division of Plastic and Reconstructive Surgery, Keck School of Medicine, University of Southern California, Los Angeles, California; Division of Plastic and Reconstructive Surgery, Keck School of Medicine, University of Southern California, Los Angeles, California

## Abstract

**Introduction:**

For years after injury, burned skin is more sensitive to UV radiation, making it susceptible to further damage and pigmentary changes. This increased risk of harm emphasizes the importance of practicing diligent sun protection. Survivors frequently experience uncertainty about which measures of sun protection are most essential and how to implement these practices in daily life, making it difficult to maintain daily routines. The purpose of this study is to better understand survivors’ knowledge of sun protection to help guide future targeted education.

**Methods:**

A cross-sectional survey was conducted at a level one trauma hospital from June 2025 to September 2025. Patients who were 18 and older and attended burn surgery outpatient clinics were included. Patients were administered a seven-item survey assessing frequency of sunscreen use on burn scars, types of sunscreens used, and factors contributing to non-adherence.

**Results:**

A total of 50 patients were surveyed. The majority of patients were male (n = 34, 68%) and English-speaking (n = 35, 70%). While 62% (n = 31) of patients reported having sunscreen at home, only 14% (n = 7) of patients applied it daily for general use. On a scale of 0-10, with 10 being the most important, 68% (n = 34) of patients rated their perceived importance of sunscreen use on burn scars between 7 and 10, indicating they considered it important. Only 28% (n = 14) of patients used sunscreen specifically on their healed burn wounds. The proportion of patients who reported using sunscreen on burn scars was significantly lower in the Spanish-speaking group (n = 1/15, 6.7%) compared to the English-speaking group (n = 13/35, 37.1%) (*p*=.046). For those who did not apply sunscreen on burn scars, the most common reasons were a lack of awareness for the need (n = 13, 36%) and the belief that it was not necessary (n = 9, 25%) (Fig. 1). Over half of the participants (n = 29, 58%) reported that their healthcare team had not informed them of sunscreen use at prior appointments. The same number of patients (n = 29, 58%) believed that they did not need additional information on sunscreen use.

**Conclusions:**

This study reveals a gap in post-burn injury care, as survivors’ perceived importance of sun protection did not translate to consistent application in practice. More broadly, over 50% of respondents indicated they had not received sunscreen counseling during prior appointments, suggesting a need for more direct and reinforced counseling strategies by burn team members to improve patient education and retention.

**Applicability of Research to Practice:**

This study exposes shortcomings in current patient counseling on sun protection after a burn and highlights systemic barriers to care which limit patient adherence to sunscreen application.

**Funding for the study:**

The contents of this abstract were developed under a grant from the National Institute on Disability, Independent Living, and Rehabilitation Research (NIDILRR grant number 90DPBU0007). NIDILRR is a Center within the Administration for Community Living (ACL), Department of health and Human Services (HHS). The contents of this abstract do not necessarily represent the policy of NIDILRR, ACL, or HHS, and you should not assume endorsement by the Federal Government.

